# Supplementary material for: The genome sequence of the Toadflax Pug, Eupithecia linariata ([Denis & Schiffermüller], 1775) (Lepidoptera: Geometridae)
Source: Wellcome Open Res. 2026 Oct 21;11:361. Originally published 2026 Jun 13. [Version 2] doi: 10.12688/wellcomeopenres.26703.2 (PMC13691353; doi:10.12688/wellcomeopenres.26703.2)
Supplement: Peer review history

# Reviewer report: Reviewer response for version 2

**DOI:** 10.21956/wellcomeopenres.30954.r175250

**Published:** 2026-10-22

**Authors:** Stuart J.E. Baird

**Affiliations:** 1 Academy of Sciences of the Czech Republic, Národní, Czech Republic

The authiors have addressed the minor issue I flagged.

Are sufficient details of methods and materials provided to allow replication by others?

Yes

Is the rationale for creating the dataset(s) clearly described?

Yes

Are the datasets clearly presented in a useable and accessible format?

Yes

Are the protocols appropriate and is the work technically sound?

Yes

Reviewer Expertise:

Admixture genomics

I confirm that I have read this submission and believe that I have an appropriate level of expertise to confirm that it is of an acceptable scientific standard.

# Reviewer report: Reviewer response for version 1

**DOI:** 10.21956/wellcomeopenres.29404.r167408

**Published:** 2026-10-14

**Authors:** Ricardo Rodriguez-de-la-vega

**Affiliations:** 1 Université Paris-Saclay, Gif-sur-Yvette, France

Comments on NHM Genome Acquisition Lab, DToL Barcoding Collective, et al., "The genome sequence of the Toadflax Pug ..."

 This collective authors' Data Note from the Darwin Tree of Life (DToL) project presents Earth BioGenome Project reference level haplotype resolved assemblies of a male specimen of *Eupithecia linariata* (commonly known as the toadflax pug). Assemblies were obtained with hifiasm on HiFi long-reads scaffolded with Arima-HiC reads, resulting in two haplotypes, the primary of which was manually curated in PretextView and HiGlass using Hi-C data to generate a chromosome level assembly, leaving the other haplotype at the scaffold level, notably quality of both assemblies meet the Earth Biogenome Project reference.

 Sample acquisition, nucleic acid extraction, library preparation and crosslinking, sequencing and assembly followed the well-established DToL's standard operating procedures, dully referenced in the Data Note. I spotted no technical issues. As common for recent (since early 2026) DToL-derived Data Notes, supporting figures include:

 - GenomeScope kmer frequency vs kmer coverage of HiFi reads,

 - Hi-C contact map of the primary assembly,

 - MerquryFK kmer multiplicity of reads and assemblies,

 - BlobToolKit snail plot of the primary assembly and

 - Coverage (minimap2 of reads on assembly) vs gc content length-scaled scatter plot with length distribution histograms.

 Although the Z chromosome is said to have been identified by mapping Merian elements, how this was done is not disclosed. In the original reference (Wright et all., 2024 in the references' list) authors used the tool syngraph (Mackintosh et al., 2023) to define the Merian elements. It is not clear to me if authors of this Data Note have followed a similar approach or have been directly use the shiny app (https://charlottejwright.shinyapps.io/busco_painter/ input: busco full_table.tsv run with --datasets_version odb10) or python code associated with the original reference (https://github.com/charlottewright/lep_busco_painter). If the Data Note gets updated, it would be good that this procedure is more clearly explained.

 I have run BUSCO 6.0.0 with -l lepidoptera --datasets_version odb10 for both assemblies. Worth noticing is that there is a perfect match between the assembled chromosomes in the manually curated primary assembly and the scaffolds in the second haplotype, both in terms of composition and with regard to location of Merian elements (i.e. the assemblies are collinear). It would be interesting to check how often this is the case among DToL or other EBGP project (e.g. Psyche also released through Wellcome Open Research).

 For the sake of convenience, I am including links to the DToL quality check and Genomes on a Tree page for this specimen/species.

 https://tolqc.cog.sanger.ac.uk/darwin/insects/Eupithecia_linariata

 https://goat.genomehubs.org/record?recordId=873508&result=taxon&taxonomy=ncbi#Eupithecia%20linariata

Are sufficient details of methods and materials provided to allow replication by others?

Partly

Is the rationale for creating the dataset(s) clearly described?

Yes

Are the datasets clearly presented in a useable and accessible format?

Yes

Are the protocols appropriate and is the work technically sound?

Yes

Reviewer Expertise:

Bioinformatics, Evolutionary Genomics

I confirm that I have read this submission and believe that I have an appropriate level of expertise to confirm that it is of an acceptable scientific standard.

## References

[1] : Inferring inter-chromosomal rearrangements and ancestral linkage groups from synteny. *bioRxiv* .2023; 10.1101/2023.09.17.558111 10.1101/2023.09.17.558111

# Reviewer report: Reviewer response for version 1

**DOI:** 10.21956/wellcomeopenres.29404.r167409

**Published:** 2026-10-08

**Authors:** Stuart J.E. Baird

**Affiliations:** 1 Academy of Sciences of the Czech Republic, Národní, Czech Republic

The genome note follows the excellent DToL template.

 I have two issues only:

 (1)

 Background

 “In Great Britain and Ireland, it is resident and common, being well distributed through much of England and Wales, more local in Scotland, rare in the Channel Islands, and recorded historically from the Isle of Man.”

 The species is also historically recorded in Ireland.

 (2)

 "The species is associated with rough chalk and limestone grassland, roadside verges, broken ground, derelict land and some urban sites. Adults fly mainly from late May to early September, and the larvae feed on Common Toadflax, first in the flowers and later in the seed capsules"

 The species is usually described as associated first with two food plants, rather than the context in which they in turn are found: Common Toadflax ( *Linaria vulgaris*) and cultivated Snapdragons ( *Antirrhinum*). I find it strange to frontload the substrate association, as the foodplant association explains not only the common name of this geometrid, but also the scarcity of the Toadflax Pug in Ireland: despite its name, Common Toadflax is extremely rare in Ireland (and likely introduced). Likewise, Toadflax is a rare site on the Isle of Man: it appears the main constraint of the species distribution is the food plant, not soils that food plants could in principle grow on.

Are sufficient details of methods and materials provided to allow replication by others?

Yes

Is the rationale for creating the dataset(s) clearly described?

Yes

Are the datasets clearly presented in a useable and accessible format?

Yes

Are the protocols appropriate and is the work technically sound?

Yes

Reviewer Expertise:

Admixture genomics

I confirm that I have read this submission and believe that I have an appropriate level of expertise to confirm that it is of an acceptable scientific standard, however I have significant reservations, as outlined above.

# Response

**Published:** 2026-10-14

**Authors:** Tree of Life Team Sanger

**Affiliations:** Wellcome Trust Sanger Institute, UK

Thank you for your helpful comments. We have corrected the text of the Background.

 The Tree of Life team
